# Impact of Six Rounds of Mass Drug Administration on Brugian Filariasis and Soil-Transmitted Helminth Infections in Eastern Indonesia

**DOI:** 10.1371/journal.pntd.0002586

**Published:** 2013-12-12

**Authors:** Taniawati Supali, Yenny Djuardi, Mark Bradley, Rahmah Noordin, Paul Rückert, Peter U. Fischer

**Affiliations:** 1 Department of Parasitology, Faculty of Medicine, University of Indonesia, Jakarta, Indonesia; 2 Global Health Programs, GlaxoSmithKline, Brentford, Middlesex, United Kingdom; 3 Institute for Research in Molecular Medicine, Universiti Sains Malaysia, Penang, Malaysia; 4 German International Co-operation (GIZ), Kupang, Indonesia; 5 Department of Internal Medicine, Infectious Diseases Division, Washington University School of Medicine, St. Louis, Missouri, United States of America; Parasite Epidemiology Research Group, Division of Clinical Epidemiology, Research Institute of the McGill University Health Centre, Canada

## Abstract

**Background:**

The lymphatic filarial parasite *Brugia timori* occurs only in eastern Indonesia where it causes high morbidity. The absence of an animal reservoir, the inefficient transmission by *Anopheles* mosquitoes and the high sensitivity to DEC/albendazole treatment make this species a prime candidate for elimination by mass drug administration (MDA).

**Methodology/Principal Findings:**

We evaluated the effect of MDA using DEC and albendazole on *B. timori* and soil transmitted helminths (STH) in a cross-sectional study of a sentinel village on Alor Island annually over a period of 10 years. Pre-MDA the microfilaria (MF) prevalence was 26% and 80% of the residents had filaria-specific IgG4 antibodies. In 2010, 34 months after the 6^th^ round of MDA, MF and antibody rates were only 0.17% and 6.4%, respectively. The MDA campaign had also a beneficial effect on STH. Baseline prevalence rates for *Ascaris*, hookworm and *Trichuris* were 34%, 28%, and 11%, respectively; these rates were reduced to 27%, 4%, and 2% one year after the 5^th^ round of MDA. Unfortunately, STH rates rebounded 34 months after cessation of MDA and approached pre-MDA rates. However, the intensity of STH infection in 2009 was still reduced, and no heavy infections were detected.

**Conclusions/Significance:**

MDA with DEC/albendazole has had a major impact on *B. timori* MF and IgG4 antibody rates, providing a proof of principle that elimination is feasible. We also documented the value of annual DEC/albendazole as a mass de-worming intervention and the importance of continuing some form of STH control after cessation of MDA for filariasis.

## Author Summary

The impact of six annual rounds of mass drug administration (MDA) using DEC combined with albendazole on brugian filariasis and soil-transmitted helminths (STH) was evaluated. Microfilaria rates of *B. timori* dropped quickly after MDA and were below 1% for 34 months after stopping intervention when the study ended. The prevalence of filarial-specific IgG4 antibodies in the community as measured by the Brugia Rapid test was about 80% before MDA and dropped slowly to about 6% at the end of the study period. MDA had also a beneficial effect on STH, especially on hookworm, but prevalence rates rebounded 34 months after cessation of MDA and quickly approached pre-control levels, while infection intensity was still reduced. Our study indicated that *B. timori* infection can be eliminated by DEC/albendazole MDA and that the anti-filarial IgG4 rate in the community significantly declines over time. While lymphatic filariasis (LF) MDA can be considered as a powerful general deworming campaign, STH infection rates rebound quickly and a supplementary control strategy is needed after LF MDA is ceased.

## Introduction

Lymphatic filariasis (LF) has been targeted by the World Health Organization for global elimination by the year 2020 [1]. During the years 2000 to 2009 the Global Program to Eliminate Lymphatic Filariasis (GPELF) has provided >2.8 billion treatments with anti-filarial drugs to a minimum of 885 million individuals living in 53 endemic countries [2], [3]. The recommended oral regimen for use in Asia is annual mass drug administration (MDA) with diethylcarbamazine (DEC, 6 mg/kg body weight) combined with albendazole (alb, fixed dose of 400 mg) [1]. We have previously published a preliminary report on the impact of two annual rounds of MDA on brugian filariasis in Alor Island in Eastern Indonesia [4]. Other studies have shown that *Bm*R1 rapid antibody test [5], [6] is a sensitive marker for detecting brugian filariasis in populations. However, more data are required to validate antibody testing as a tool for monitoring the impact of MDA on filariasis in populations.

Studies on bancroftian filariasis in Egypt concluded that five rounds of MDA may have been sufficient to eliminate the infection in most implementation units in that country [7]. Declines in rates of infection markers such as circulating filarial antigenemia and microfilaremia were accompanied by diminished rates of anti-filarial IgG4 antibodies in school children. Similar findings were reported after three rounds of MDA in Papua New Guinea, where antibody prevalence decreased faster in children than in adults [8]. Anti-filarial antibodies are a marker for past, present or exposure to filarial infections. Antibody clearance tends to occur faster after treatment in children than in adults, since children tend to have shorter term exposure and lighter infections. Therefore, adults may have persistent antibodies years after effective therapy [9], [10]. Less is known about antibody clearance after treatment of brugian filariasis, and research is needed to determine the dynamics of this clearance with different antibody assays.

Mass drug administration of DEC combined with alb has an additional beneficial effect in reducing prevalence and intensity of infection with intestinal helminths such as *Ascaris lumbricoides*, hookworms and *Trichuris trichiura* [4], [11], [12]. Unfortunately, most studies have evaluated the effect of MDA on intestinal helminths after only one or two treatment rounds in school-aged children, and data on the impact of multiple rounds after population-based MDA used in filariasis elimination programs are lacking.

Pilot studies in 2001 detected a high prevalence of *B. timori* infection and filariasis-associated morbidity in the highlands of Alor island [13]. We initiated treatment trials and worked with local health officials to develop an MDA program on the island. We reported the results of the first two rounds of MDA in prior publications [4], [14], [15]. The objective of the present paper is to evaluate the impact of six annual rounds of MDA on brugian filariasis and on soil-transmitted helminths (STH) infections in a sentinel village on Alor and also report the results collected over 3 years following the last round of MDA.

## Methods

### Study area

The study was performed in Mainang village (population in 2002 approximately 1,500) on Alor island (East Nusa Tenggara Timor, Indonesia). Details of the study site have been published elsewhere [4], [13]. Conditions in Alor and in Mainang changed over the course of the study. For example, the island received considerable financial support following a major earthquake in 2004 which improved infrastructure and living conditions. Bed net use has increased and the hygienic conditions have improved during the study period. However, Alor district remains one of the poorest districts of Indonesia.

### Sample collection

**Figure 1:**
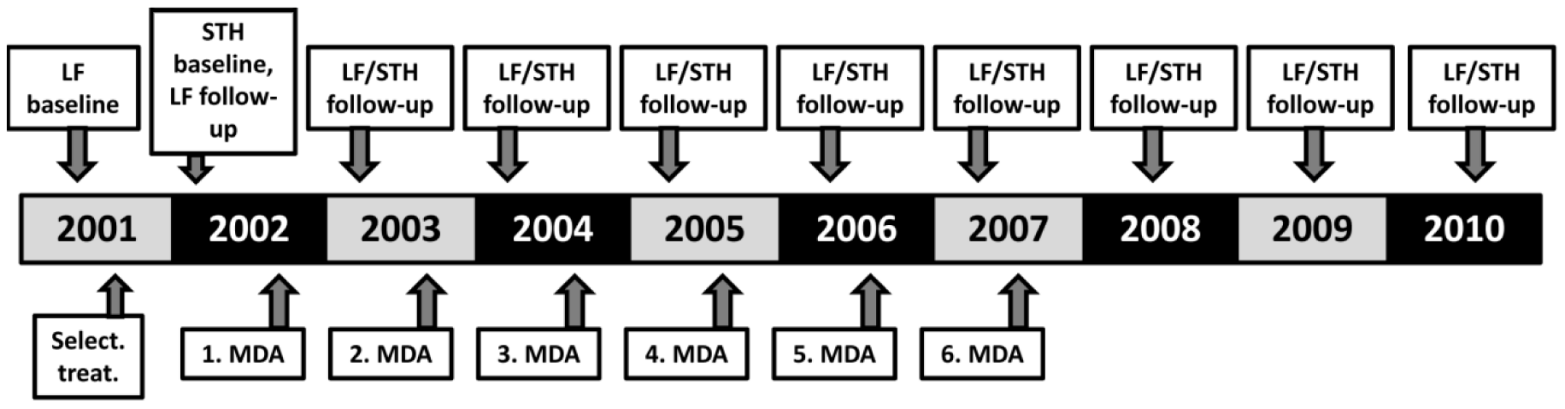
Schematic overview of the study design. In 2001 microfilaria and anti-filarial IgG4 baseline surveys were performed and MF positive and lymphoedema patients were selectively treated. Baseline data on STH infections was collected in 2002, and the village was re-examined for filarial infections at that time just prior the first round of MDA with diethylcarbamazine plus albendazole. Subsequent post-MDA surveys were performed 9 to 11 months after each round of MDA. MDA was continued until 2007, when the last (6^th^) round of MDA was provided and annual follow-up surveys were continued until 2010.

Over the 10 year study period (Fig. 1) the study population of the three study sectors of the village increased from about 1,500 to about 1,800. Annual surveys collected samples from 600–750 residents, which comprised 33%–50% of the eligible population. Children younger than 3 years and severely ill persons were considered not eligible and excluded from the surveys. Almost all residents were examined at least once over the study period, while most individuals were examined twice or three times. However, only about 20% of the population participated in all 10 surveys. The study population and the sample collection procedure were described in detail in earlier reports [4], [13]. Briefly, sex, age and name were noted; after a brief clinical examination 3 ml venous blood was collected between 7.00 p.m. and 11 p.m. One labeled stool container was provided to each individual participating in the blood collection, and these were collected one day later by local health workers.

### Ethics statement

The study was approved by the ethical board of the Faculty of Medicine, University of Indonesia, Jakarta and the institutional review boards at Bernhard Nocht Institute for Tropical Medicine in Hamburg, Germany, as well as at Washington University School of Medicine in St. Louis, USA. Since written consent is not consistent with cultural norms on Alor island, oral informed consent was obtained from all adults or, in case of children, from their parents. The ethical board of the University of Indonesia and the institutional review boards in Germany and the USA approved the use of oral consent. The participant's oral consent was noted on the survey questionnaire.

### Mass drug administration

Community-based MDA using a single dose of DEC and alb was performed by the local District Health Authority. The local primary health care center (“Puskesmas”) trained a number of villagers (cadres) that were responsible for distributing anthemintics to 100 to 200 residents in their neighbourhood by directly observed treatment. Pregnant or breastfeeding women, children younger than two years of age, or persons suffering from acute or severe illnesses were not eligible for treatment. In most cases cadres knew which community members were ineligible (pregnant or lactating women), but they used a short interview to assess eligibility for others. Medications were dosed based on age rather than on weight, as previously described [4]. The District Health Authority reported coverage rates (number of distributed doses per number of residents) between 75% and 85% for all years of MDA (Paul Manoempil pers. commun.). In addition, an independent survey of compliance in five villages on Alor in the first year of the MDA program documented a compliance rate of about 88% [16]. Compliance with MDA was defined as the percentage of the eligible population that reported that they had swallowed the drugs. MDA was discontinued following the treatment round in 2007 (Fig. 1), because MF rates had fallen below 1% in the previous 2 years and because no funds were available at that time to continue MDA or to perform a more extensive assessment according to the WHO guidelines at that time [17].

### Assessment of microfilariae and helminth eggs

Microfilaraemia was determined by membrane filtration of 1 ml of night blood collected in EDTA as previously described [4].

A single stool sample was examined for intestinal helminths from each individual surveyed. The Harada-Mori hatching test was used to detect living hookworm larvae as described previously [4]. Based on larval morphology and PCR, the predominant hookworm species in Alor was found to be *Necator americanus* (Supali and co-workers, unpublished results). Part of the stool samples was preserved in the field using 4% formaldehyde and examined in the laboratory in Jakarta using the formalin/ether-enrichment method within 2 months of collection.

The Kato-Katz method was used to assess STH infections in the baseline survey in 2002 and again in 2009, 22 months after the 6^th^ (and final) round of MDA (Fig. 1). A single Kato Katz smear of 41 mg of stool was performed to determine STH egg counts and to classify egg densities according to WHO guidelines as light, moderate and heavy intensity infection [18]. Stool samples were examined (Kato Katz, Harada-Mori) or preserved (formalin/ether-enrichment) within 18 h of distribution of stool containers. For tests other than the Kato-Katz, the numbers of helminth eggs or hookworm larvae were arbitrarily scored as follows: low density (1–10 eggs per slide or 1–50 hookworm larvae per plastic bag containing approximately 0.5 g stool), moderate density (11–100 eggs or 51–500 larvae) or high density (more than 100 eggs or 500 larvae).

### Detection of anti-filarial IgG4 antibodies

Specific IgG4 antibodies were detected using the dipstick version of the Brugia Rapid test (Malaysian Bio-Diagnostics Research Sdn Bhd, Bangi, Malaysia). Tests were performed in the field using 25 µl of plasma as previously described [19].

### Statistical analysis

The total number of subjects included in the surveys was calculated using a 95% confidence level and a confidence interval of less than 3%. The chi-square test was used to assess the significance of differences in infection prevalence rates between different groups or between study periods, while the Mann-Whitney U test was used to assess infection intensity data. A *p* value is regarded as significant if less than 0.05. All statistical analyses were done using the SPSS software package (IBM).

## Results

### 1. Effects of MDA on microfilaremia

**Figure 2:**
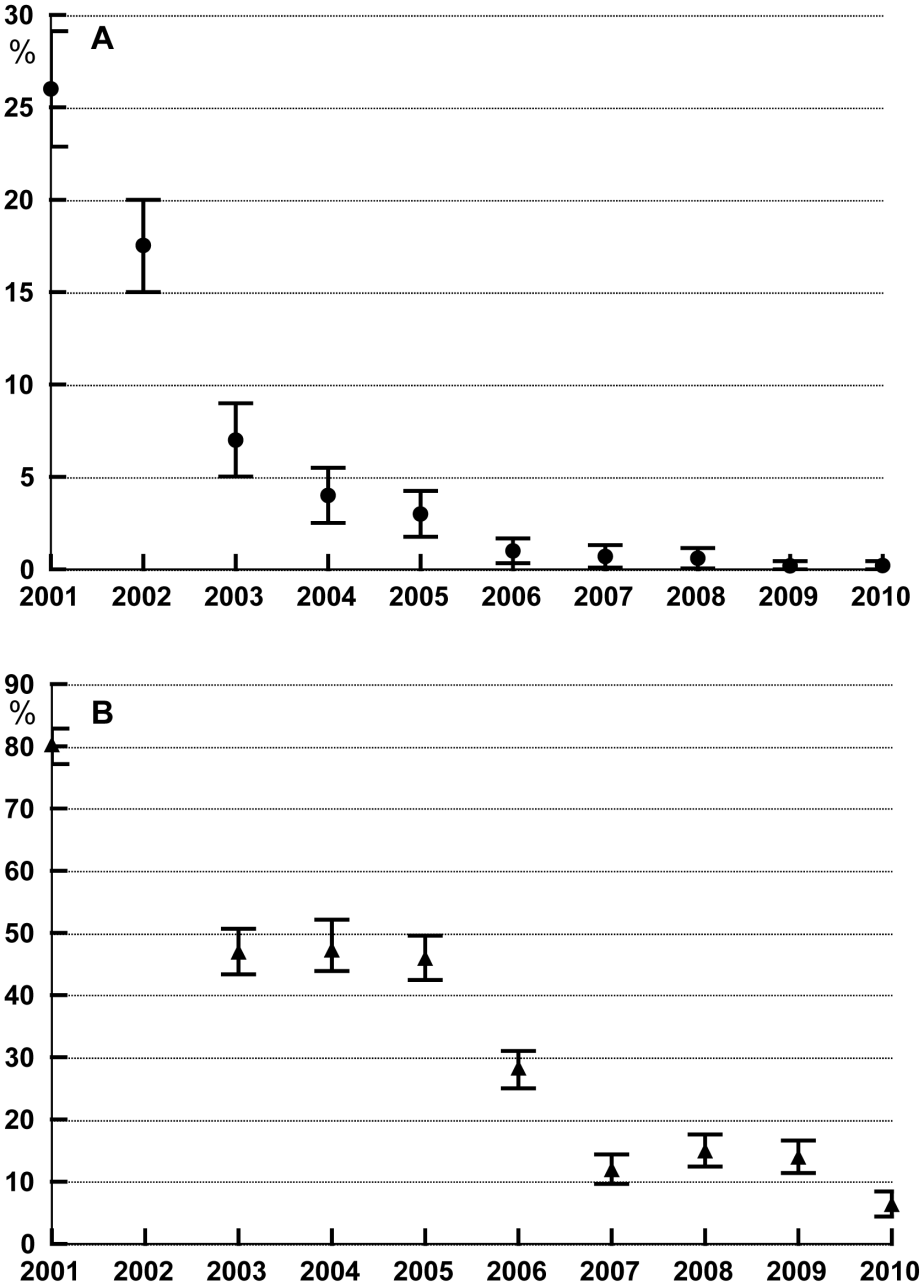
A Prevalence rates with 95% confidence intervals for *Brugia timori* microfilaraemia in Mainang village before and after MDA with DEC/albendazole. Selective treatment of microfilaraemics and lymphedema patients was performed after the baseline survey in 2001. The first round of MDA was distributed following the survey in 2002, and the last (6^th^) round of MDA was distributed after the 2007 survey was performed. MDA coverage rates ranged between 75 and 85%. **B** Prevalence rates with 95% confidence intervals for anti-filarial antibodies as assessed by the Brugia Rapid test. Since only selective treatment was performed in 2001, no Brugia Rapid tests were performed on the samples collected in 2002.

Changes in microfilaremia prevalence are shown in Figure 2A. MF rates fell from a baseline of 26% to less than 1% after round 4, and this decrease was maintained for the duration of the study including the period following cessation of MDA. Only 5 MF-positive individuals were detected among 737 individuals tested one year after the 6^th^ round of MDA. Three females (18, 19 and 47 years old) and two males (19 and 26 years old) had microfilaria densities of 1356, 63, 6, 66, and 311 MF/ml respectively. The two men were long-term visitors from another village who reported that they had never received treatment. The three females had participated in the pre-treatment blood collection, and their MF counts at that time were 88, 2456 and 333MF/ml, respectively. Two of the women had not regularly participated in the surveys, but one was examined annually and was found to be MF-positive during all examinations. In 2009 and 2010, 22 and 34 months after the 6^th^ and last round of MDA, we examined a total of 668 and 598 persons, respectively. Only one woman was found to be MF-positive with 192 MF/ml and 115 MF/ml, respectively. It was the same young lady who had been found to be MF-positive all the years before. At first she claimed to have regularly participated in MDA, but later she admitted that she had not taken the medicines after the first treatment round because she had experienced adverse events that included high fever and severe headache.

### 2. Effects of MDA on anti-filarial IgG4 antibodies

**Figure 3:**
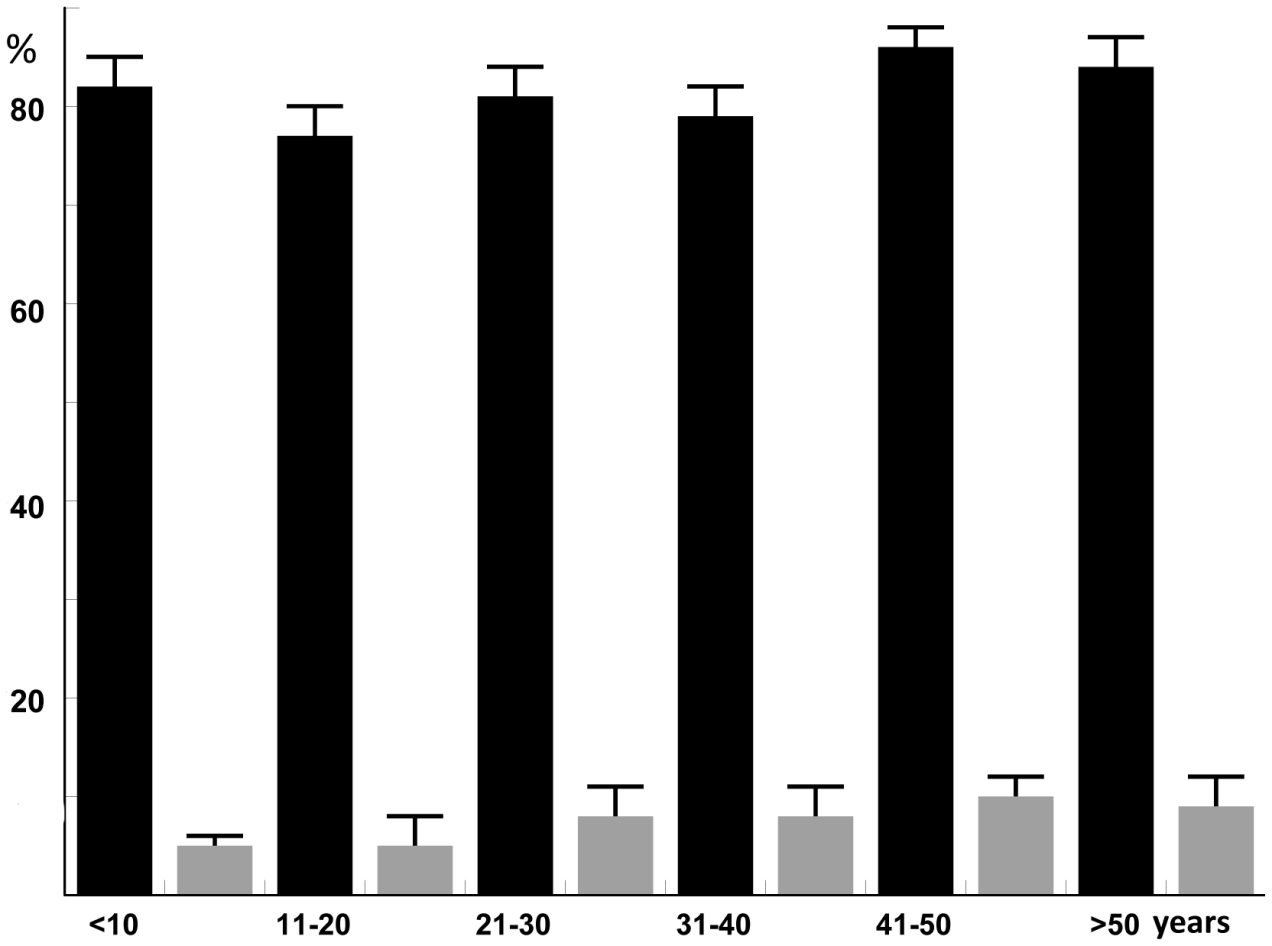
Prevalence rates with 95% confidence intervals for antifilarial antibodies by age group as assessed by the Brugia Rapid test. Baseline data for 2001 (prior to MDA) are shown with black bars should be compared with data from 2010 (black bars) (about 34 months after the 6th and final round of MDA).

We used the Brugia Rapid dipstick test as a marker for ongoing or prior infection, because there is no adult worm antigen detection test for *Brugia spp*. This test employs a recombinant *B. malayi* antigen (*Bm*RI) that has been extensively studied [19]. Antibody test results are shown in Figure 2B. Briefly, the antibody rate decreased from 80% at baseline to a rate of 6.4% in 2010. There was no significant difference between antibody prevalence rates in male and female subjects all through the study. As reported previously, antibody rates were high (about 80%) in all age groups in 2001 [6]. In 2010 the antibody rates had strongly declined. The rates varied with age (4.9% for subjects less than 21 years of age versus 8.7% for older subjects) (Fig. 3). However, this difference between the two age groups was not significant. WHO now recommends transmission assessment surveys (TAS) to support decisions to stop MDA. The TAS are designed to systematically sample school-aged children within an evaluation unit [20]. In the absence of a rapid antigen test for *Brugia*, antibody rates in school-aged children are used as the indicator for TAS. Therefore, we analysed the decline of the filarial antibody rate as determined by Brugia Rapid in pre-school and primary school-aged children (Table 1). While the positive test rate for Brugia Rapid in children aged between 3 and 10 years dropped rapidly following MDA, the prevalence and the upper 95% confidence limit dropped below 5% and 10%, respectively, for the first time in 2009, 22 months after the last round of MDA. In 2009 the majority of children aged between 3 and 10 years were born after the initiation of MDA, but many of these children might have been exposed to *B. timori* during the early years of the MDA program.

**Table 1:** Prevalence rates with 95% confidence intervals for anti-filarial antibodies based on the Brugia Rapid test in children aged between 3 and 10 years of age prior to MDA (2001) and after one or more rounds of MDA.

Year	N examined	% Brugia Rapid Positive	95% CI
2001	120	82.0	75.1–88.8
2003	243	39.1	32.9–45.2
2004	202	41.1	34.3–47.9
2005	164	22.6	16.2–29.0
2006	138	13.0	7.4–18.6
2007	119	6.7	2.2–11.2
2008	115	13.9	7.4–20.2
2009	103	3.9	0.2–7.6
2010	116	4.9	0.6–8.0

The last round of MDA was performed in 2007. Since only selective treatment of MF carriers was performed in 2001, no Brugia Rapid tests were performed on the samples collected in 2002.

The relationship between antibody and MF rates varied considerably during the study period (compare Fig. 2A and B). The antibody to MF ratio was 3.1 at baseline, but increased dramatically after MDA. This means MF rates fell more rapidly after MDA than antibody rates. For this reason, it is not possible to use antibody rates to estimate MF prevalence rates following MDA.

### 3. Prevalence of soil transmitted helminths

**Figure 4:**
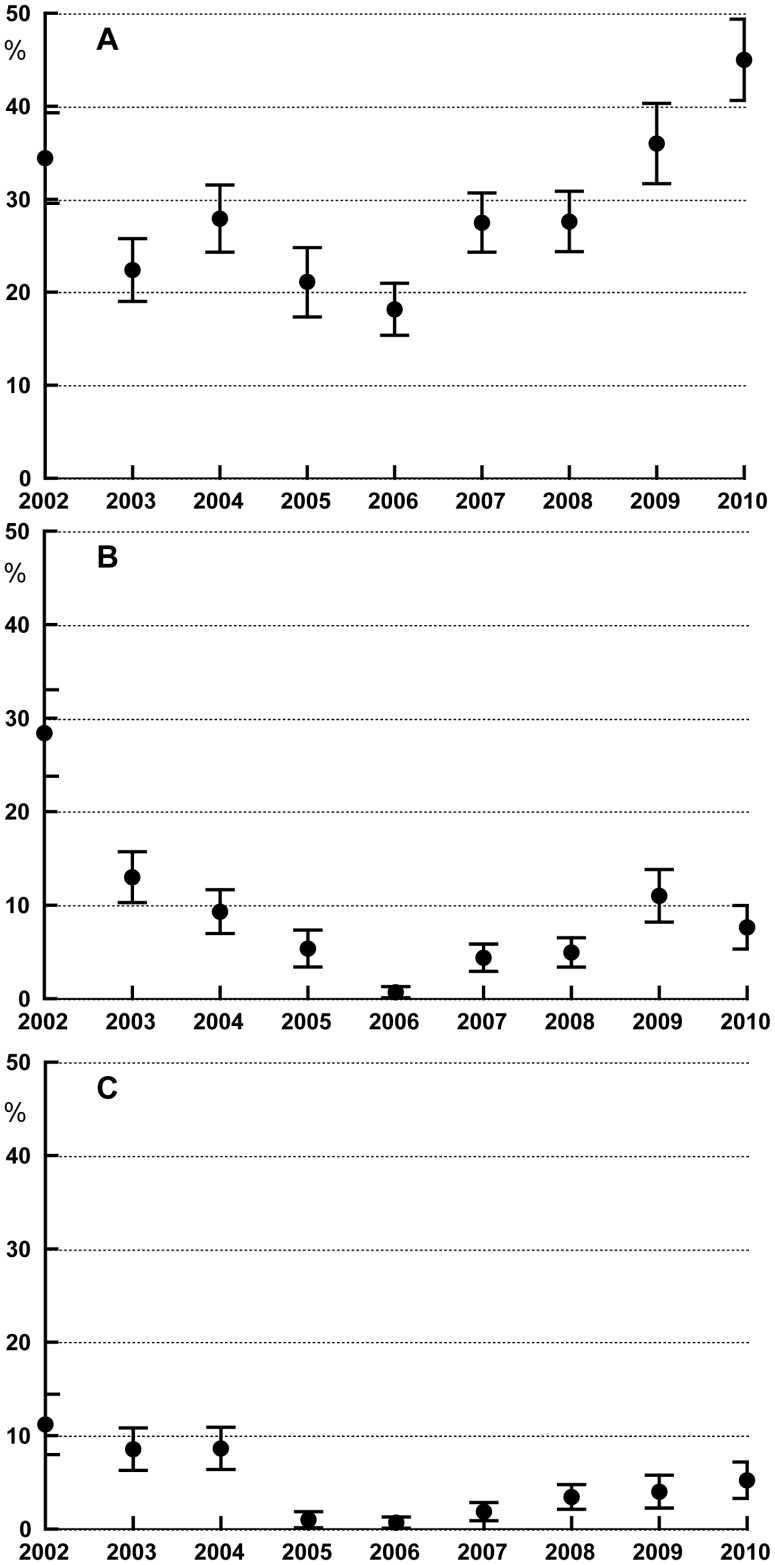
Prevalence rates with 95% confidence intervals for STH infections in Mainang village before and after MDA with DEC/albendazole. The first round of MDA was performed just after this the 2002 survey. The 2007 STH data were from a survey that was performed just prior to the last (6^th^) round of MDA. **A** *Ascaris lumbricoides*. **B** Hookworm (mainly *Necator americanus*) and **C** *Trichiuris trichiura*.

*Ascaris* prevalence rates varied considerably during the study period (Fig. 4A). The lowest prevalence of 18% was detected in 2006 after 4 rounds of MDA. In 2010, 34 months after the last round of MDA, the prevalence (45%) was significantly higher than the baseline prevalence (34%) prior to MDA (p<0.01).

In contrast, the hookworm prevalence rates decreased from 28% at baseline to 13% after the first round of MDA and continued to decrease to 0.7% in 2006 (Fig. 4B). The hookworm prevalence rates were determined all through the study period by two independent methods, formalin/ether enrichment and the Harada Mori hatching test, which increases the overall sensitivity. Hookworm rates rebounded to 7% at 34 months after the last MDA.

The first two rounds of MDA had little impact on the *Trichuris* prevalence rate (baseline 9.4%), but this decreased dramatically to less than 1% after the third round (Fig. 4C). The lowest rate (0.7%) was observed after the last round of MDA in 2006; this slowly increased after MDA ended to 7% in 2010. Interestingly, STH infection rates were similar for male and female subjects (p>0.05%), and there were no significant differences in age specific infection rates in 2002 prior to MDA as well as in 2010 (p>0.5%).

### 4. Intensity of soil transmitted helminth (STH) infections

Infection intensities (by Kato-Katz) were assessed before MDA in 2002 and in 2009, 22 months after the 6^th^ round of MDA. As an aside, infection intensities determined by Kato Katz were generally consistent with informally scored infection intensities obtained by the ether enrichment and Harada Mori methods.

**Figure 5:**
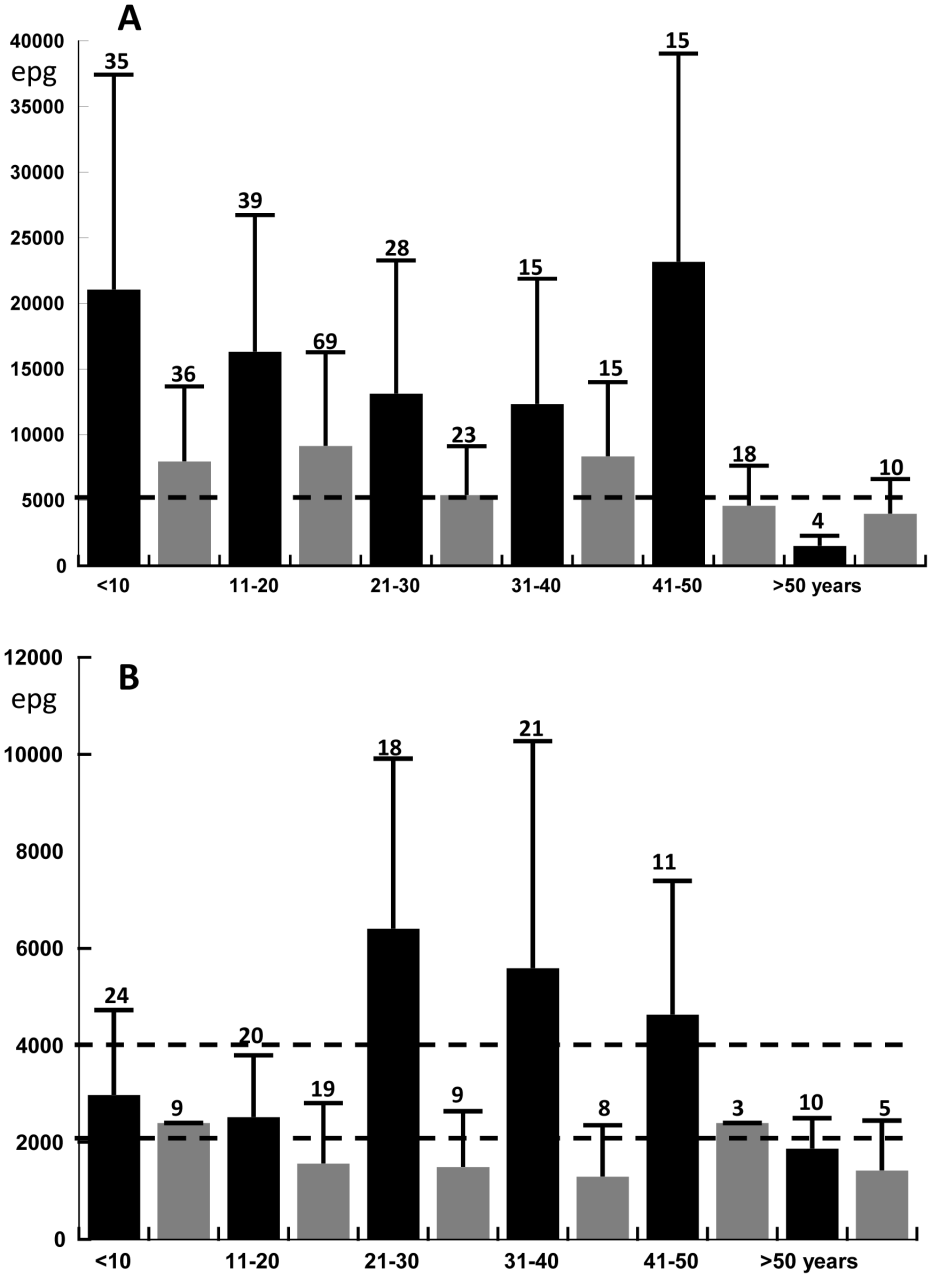
Geometric mean egg counts per gram of stool (as assessed by a single Kato-Katz smear) for individuals with STH infections in Mainang village in 2002 (before the 1^st^ round of MDA, black bars) and in 2009 (22 months after the 6^th^ and final round of DEC/albendazole MDA, gray bars). The data are shown by age group. Error bars indicate the 75 percentiles, and the broken lines represent thresholds for light and moderate infection intensities according to WHO guidelines. The number on top of each column represents the number of positive individuals tested per age group. **A** *Ascaris lumbricoides*. **B** Hookworm (mainly *Necator americanus*).

MDA has shifted the frequency distributions of STH intensities so that fewer subjects had heavy infections in 2009 than in 2002, and these data are summarized in Figure 5. For example, infected subjects in 5 of the 6 age groups had geometric mean of *Ascaris* infection intensities in the moderately high range (between 5,000 and 49,000 eggs per gram [epg] according to WHO criteria) in 2002, and the geometric mean egg count in all infected subjects was 15,000 epg. In 2009, three age groups had geometric mean egg counts in the moderately high infection intensity range; the geometric mean count for all infected subjects was 6,500 epg, which represented a 57% reduction from the baseline value (Fig. 5A).

Similar results were obtained for intensities of hookworm infection (Fig. 5B). Adults aged 21 to 50 years with hookworm infections had geometric mean egg counts in the high range (>4,000 epg) in 2002, while children and adolescents had geometric mean counts in the moderately high range (2,000–3,999 epg), while other age groups had moderate mean infection intensities. In 2009, no age group had geometric mean epg in the high range, while moderate range geometric mean counts were only seen in children less than 10 years of age and in adults in the 41–50 years age group. Geometric mean egg counts for *T. trichiura* in 2009 were not significantly different from those in 2002, and they tended to be low in all age groups at both time points (range 48–164 epg). However, only relatively few *Trichuris* infections were assessed in 2009 quantitatively.

## Discussion

We have studied the dynamics of helminth infection during and for up to 34 months after six rounds of annual MDA using DEC combined with albendazole in one sentinel village in eastern Indonesia. The MF rate fell to less than 1% in 2006, and this declined further to 0.17% in 2010 when the study ended. MDA rapidly reduced MF counts in the population. We would have detected fewer MF carriers if we had used thick blood smears for MF detection according to WHO guidelines instead of the more sensitive membrane filtration method [21]. In 2009 and 2010 only a single MF positive individual was detected. This individual is an example for systematic non-compliance with treatment because of the fear of adverse events. The contribution of a single MF positive person to the transmissions cycle appeared to be negligible because no new MF positive individuals were found. Anti-filarial antibody rates in the general population fell from 80% in 2001 to 6.4% in 2010. The antibody rate in children and young adults less than 21 years of age in 2010 was 4.9%. These results suggest that MDA interrupted transmission of *B. timori* infection in Mainang village.

Since the MF rate in 2006 (after the 4^th^ round of MDA) was below 1% and remained below this threshold for the next 2 years, the District Health Authority decided to stop MDA in 2008 due to financial constraints. When external funding became available, a TAS was performed in the entire district (Alor and Pantar islands) in 2009 and in 2011 according to WHO guidelines [17], [20]. The district passed the TAS in both years, and this suggests that the decision to stop MDA in 2008 was valid (Supali and co-workers, unpublished results).

Analysis of anti-filarial IgG4 antibody rates over the 10 year study period of the study showed that this rate has declined more slowly than the MF rate following MDA. Current guidelines recommend testing for antibody in children as a tool for detecting ongoing transmission of brugian filariasis [10], [21]. Our study showed that IgG4 antibody rates as determined by Brugia Rapid declined in children as well as in adults and eventually reached very low levels some nine years after initiation of MDA and after six annual rounds of MDA. The fact that a few children aged ≤10 years in 2010 had positive antibody tests can be explained by the high antibody rate in young children in 2001 and the fact that it takes several rounds of MDA to interrupt transmission of *B. timori* [6]. The observation that it took 6 rounds of MDA and additional 22 months until the filarial antibody rate as determined by the Brugia Rapid test dropped below 5% (upper 95% confidence limit 10%) in children aged between 3 and 10 years suggests that the antibody rate is a sensitive marker for previous exposure to *B. timori*. While all examined children in that age group were constantly MF negative following the first two rounds of MDA, the antibody prevalence dropped quickly but remained higher than 2% for the entire study period. A target of 2% antigen prevalence is used for TAS in *W. bancrofti* areas [20]. It is possible that a higher threshold of antibody prevalence is needed for TAS in *Brugia* areas. However, Alor district as evaluation unitis endemic for *B. timori* and *W. bancrofti* and passed the TAS in 2009 and in 2011 (one year after conclusion of the present study) using antibody and antigen tests (Supali et al, unpublished results) despite the fact that one primary school in Mainang village was included in the surveys,

In order to use antibody rates in the general population as a marker for evaluation of an MDA program, frequent follow-ups are necessary to assess the decline of antibody prevalence over time. It is likely that antibody rates would increase if transmission resumes in the area, but this can only be assessed if longitudinal antibody data are available for the study area. Moreover, the Brugia Rapid test has already been employed to assess the success *Brugia malayi* elimination from parts of the Republic of Korea [22].

The benefits of MDA using DEC combined with albendazole beyond LF elimination have been extensively discussed previously [23], [24]. However, field data on the effects of MDA on STH are limited since most programs in Africa use ivermectin instead of DEC, and results are often available only for one or two rounds of MDA. In addition results are often not as clear as expected. For example in an area with low STH prevalence and relatively low compliance to MDA in Sri Lanka, it was concluded that four rounds of MDA had little effect on STH infections in school children [25]. In India only a single round of MDA showed reduction of STH infections in school children at several time points following treatment [26]. Prior studies have shown that individuals become rapidly re-infected with STH and that re-infection rates for *Ascaris* are the highest [27]. Since rapid re-infections are a major problem for STH, evaluation of the effect of MDA on STH should be done probably more frequently or closer to the next treatment date. Modeling studies have predicted that LF elimination can be achieved more rapidly by employing twice yearly MDA, while reducing the overall program costs [28]. Twice yearly MDA could help to prevent re-infection of STH and should, therefore, show a stronger benefit for STH reduction. On the other hand the present study also shows an effect of MDA on STH using annual follow-ups.

Unfortunately, when MDA for LF was stopped in 2008, the district government had no school- or community-based STH deworming program in place to help preserve the beneficial effect that MDA had had on STH. While it was relatively easy for the district government to obtain external funds for LF elimination (since that was an attractive new public health program), it was not possible for the district to obtain support for routine de-worming. At the time the discussion about integration of control and elimination programs for the different NTDs was just starting.

Taken together our study has provided evidence that *Anopheles*-transmitted *B. timori* can be eliminated by MDA alone, and that antibody prevalence assessed by Brugia Rapid in the population decreases after MDA but at a slower pace than MF prevalence. Our data also show that MDA for LF with DEC plus albendazole is a mass de-worming program that reduces prevalence rates for hookworm and *Trichuris* and also reduces STH infection intensities. However, the study also showed STH rates rebounded quickly after MDA was discontinued. Further work is needed to develop strategies and guidelines for controlling STH in communities following suspension of MDA for LF.

## Supporting Information

Checklist S1 STROBE checklist.
(DOC)
